# THE EFFECT OF DUAL ELIGIBILITY ON IMPROVEMENT IN MOBILITY: VARIATION BY PATIENTS’ PRIMARY DIAGNOSIS

**DOI:** 10.1093/geroni/igz038.1856

**Published:** 2019-11-08

**Authors:** Molly W Vaughan, Ila H Broyles, Melvin J Ingber, Lauren A Palmer, Tara McMullen, Karyn K Anderson, Anne Deutsch

**Affiliations:** 1 RTI International, Waltham, Massachusetts, United States; 2 RTI International, Research Triangle Park, North Carolina, United States; 3 RTI International, Rockville, Maryland, United States; 4 Centers for Medicare & Medicaid Services, Baltimore, Maryland, United States; 5 RTI International, Chicago, Illinois, United States

## Abstract

Dually eligible individuals (i.e., eligible for Medicare and Medicaid) often have worse health and greater functional limitations than patients eligible for Medicare only. For dually eligible patients receiving inpatient rehabilitation services following a major illness or injury, improvement in function may be lower than for Medicare-only patients. To our knowledge, this is the first study to examine the relationship of dual eligibility on improvement in mobility for inpatient rehabilitation facility (IRF) patients by 13 primary diagnosis groups (e.g., Stroke, Amputation). Data was collected on the IRF-Patient Assessment Instrument at admission and discharge for all IRF patients discharged during 2017 (N = 428,631). A generalized linear model was run for each primary diagnosis group to examine the effect of dual eligibility on change in mobility during an IRF stay, adjusting for sociodemographic factors, clinical factors, and comorbidities. The proportion of patients who were dually eligible varied among primary diagnosis groups (9.6% for Hip/Knee Replacements, Fractures and Multiple Trauma to 21.7% for Amputation). Compared to patients who were non-dually eligible, dually eligible patients had lower improvement in mobility across all 13 diagnostic groups. The strongest effect of dual eligibility on lower improvement in mobility was among patients with hip and/or knee replacements (β: -1.99, p<0.001) and patients with non-traumatic spinal cord dysfunction (β: -1.83, p<0.001). This research indicates that dually eligible patients may have worse functional mobility outcomes than non-dually eligible patients for some IRF primary diagnosis groups, and these patients may need additional support after discharge.

